# Longitudinal analysis of risk factors associated with severe acute respiratory coronavirus virus 2 (SARS-CoV-2) infection among hemodialysis patients and healthcare personnel in outpatient hemodialysis centers

**DOI:** 10.1017/ash.2022.269

**Published:** 2022-07-21

**Authors:** Sumanth Gandra, Tingting Li, Kimberly A. Reske, Kate Peacock, Karl G. Hock, Silvana Bommarito, Candace Miller, Henry Stewart, Na Le Dang, Christopher W. Farnsworth, Margaret A. Olsen, Jennie H. Kwon, David K. Warren, Victoria J. Fraser, for the CDC Prevention Epicenters Program

**Affiliations:** 1 Division of Infectious Diseases, Washington University School of Medicine, St Louis, Missouri; 2 Division of Nephrology, Washington University School of Medicine, St Louis, Missouri; 3 Department of Pathology and Immunology, Washington University School of Medicine, St Louis, Missouri

## Abstract

In this prospective, longitudinal study, we examined the risk factors for severe acute respiratory coronavirus virus 2 (SARS-CoV-2) infection among a cohort of chronic hemodialysis (HD) patients and healthcare personnel (HCPs) over a 6-month period. The risk of SARS-CoV-2 infection among HD patients and HCPs was consistently associated with a household member having SARS-CoV-2 infection.

Patients with end-stage renal disease (ESRD) on chronic hemodialysis (HD) are at increased risk of severe disease and death from severe acute respiratory syndrome coronavirus 2 (SARS-CoV-2) infection.^1^ Despite vaccination, chronic HD patients are at risk for breakthrough SARS-CoV-2 infection due to attenuated^2,3^ and receding immunological response to vaccines.^4–6^ Several studies have examined the risk factors associated with SARS-CoV-2 infection among chronic HD patients; however, these were cross-sectional studies without longitudinal follow-up data.^7–10^ Longitudinal data are valuable for identifying modifiable risk factors and devising strategies to prevent SARS-CoV-2 infection among HD patients. In this prospective, longitudinal study, we examined the risk factors for SARS-CoV-2 infection among a cohort of chronic HD patients and healthcare personnel (HCP) working in 3 outpatient HD units over a 6-month period.

## Methods

This study was conducted in 3 outpatient HD units at the Washington University School of Medicine (WUSM) in St Louis, Missouri, and it was approved by the WU Human Research Protection Office. Adult patients on in-center HD and dialysis-center HCP were enrolled after giving informed consent, as described previously.^11^ A survey was administered either electronically or in-person at 3 different times (September 2020, December 2020, and March 2021) to patients and HCP to assess their household characteristics, personal behaviors and prevention activities, SARS-CoV-2 exposures, and history of SARS-CoV-2 infection since March 1, 2020.

Blood was also collected at these 3 times to assess SARS-CoV-2 infection status. Previous SARS-CoV-2 infection was assessed using the Abbott SARS-CoV-2 IgG serologic assay that targets the viral nucleocapsid protein (Abbott, Abbott Park, IL). The resulting unit for the SARS-CoV-2 IgG assay is the index (ie, signal/calibrator or S/C). An S/C value ≥1.40 was considered positive.

Electronic medical records of patients were reviewed at each survey and blood draw to obtain relevant clinical information, including dates of SARS-CoV-2 PCR test(s), hospitalizations due to SARS-CoV-2 infection, and symptoms. Comorbid conditions were reviewed at enrollment. The electronic medical records of HCP were not reviewed. All data were entered into a REDCap database.

We defined a SARS-CoV-2 case as having either a history of positive PCR test documented in the medical record or a positive IgG nucleocapsid serology result prior to each survey. For HCP, we only considered positive IgG serology to define a SARS-CoV-2 case because we did not review HCP medical records. Univariate analyses of patient characteristics among SARS-CoV-2 cases and noncases were performed using the Fisher exact test or the χ^2^ test for categorical variables and the Mann Whitney U test for continuous variables. Statistical analyses were performed using SAS version 9.4 software (SAS Institute, Cary, NC), and *P* < .05 was considered statistically significant.

## Results

**Fig. 1.**
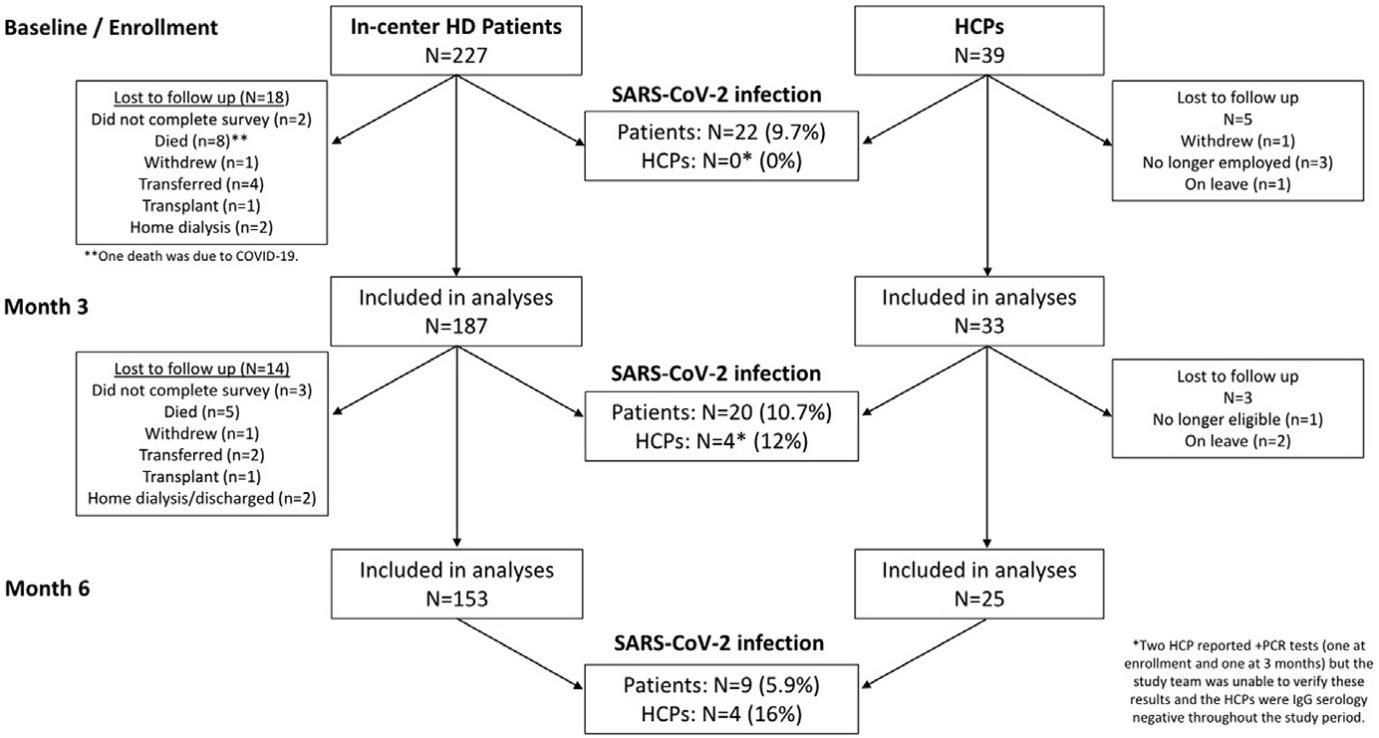
Distribution of SARS-CoV-2 infection cases among HD patients and HCPs at baseline, 3 months and 6 months.

Among the 3 HD units, 227 patients and 39 HCP were enrolled in the study (Fig. 1 and Supplementary Tables 1 and 2). At baseline, 22 (9.7%) of 227 patients had evidence of prior SARS-CoV-2 infection and none of the HCP had a SARS-CoV-2 infection. At the 3-month follow-up, 20 (10.6%) of 187 patients and 4 (15.2%) of 33 HCP had SARS-CoV-2 infection between the baseline and the 3-month follow-up. At the 6-month follow-up, 9 (5.8%) of 156 patients and 4 (16%) of 25 HCP had SARS-CoV-2 infection between the 3- and 6-month follow-ups. The cumulative incidence of SARS-CoV-2 infection among HD patients was 22.9% (52 of 227), which was 52 (26.5%) of 196 but excluding those lost to follow-up. The cumulative incidence of SARS-CoV-2 infection among HCP was 20.5% (8 of 39), which was 8 (25.8%) of 31 but excluding those lost to follow-up.

The baseline univariate analyses of risk factors associated with SARS-CoV-2 infection were published previously.^11^ In the analysis of the 3-month and 6-month surveys, SARS-CoV-2–infected cases were significantly more likely to have had a household family member with SARS-CoV-2 infection (3-month survey, 40% vs 4.2%; *P ≤* .001; 6-month survey, 11.1% vs 2.8%; *P* = .029) and to have had a household family member asked to quarantine because of exposure to SARS-CoV-2 (6-month survey, 33.3% vs 2.1%; *P ≤* .001) (Table 1). Additionally, for the 3-month survey, SARS-CoV-2–infected patients reported significantly less adherence to social distancing outside the dialysis units (50% vs 81.4%; *P ≤* .004). The only risk factors for SARS-CoV-2 infection identified among HCP were household exposure (3-month survey, 80% vs 14.3%, *P* = .009; and 6-month survey, 75% vs 4.8%; *P* = .007) and having a household member instructed to quarantine (6-month survey, 100% vs 0%, *P ≤* .001) (data not shown).

**Table 1.** Univariate Analysis of Characteristics of HD Patients Who Tested Positive or Negative for SARS-CoV-2 Infection at the 3-Month and 6-Month Follow-Up

Survey Variable	3-Month Survey (N = 187)			6-Month Survey (N = 153)		
	SARS-CoV-2 Case (N = 20, 11%)^a^	Non-case (N = 167, 89%)	*P* Value	SARS-CoV-2 Case (N = 9, 6%)^a^	Noncase (N = 144, 94%)	*P* Value
**Hemodialysis unit**						
Facility A	9 (45.0)	76 (45.5)	Reference	3 (33.3)	66 (45.8)	Reference
Facility B	9 (45.0)	71 (42.5)	.91	5 (55.6)	60 (41.7)	.42
Facility C	2 (10.0)	19 (11.4)	.89	1 (11.1)	17 (11.8)	.83
Changed transportation in prior 3 mo	3 (15.0)	12 (7.2)	.21	1 (11.1)	11 (7.6)	.53
Changed residence in prior 3 mo	2 (10.0)	11 (6.6)	.63	1 (11.1)	9 (6.3)	.47
**Demographics**						
Age, median y [IQR]	59.5 [50.5–65.5]	60 [49–69]	.31	60 [57–74]	61 [49.5–69]	.71
Sex, male	15 (75.0)	77 (46.1)	.020	3 (33.3)	71 (49.3)	.50
Race, Black	18 (90.0)	147 (88.0)	>.99	9 (100.0)	127 (88.2)	.60
BMI, median kg/m^2^ [IQR]	29.0 [27.0–33.1]	29.3 [25.2–36.5]	.88	38.0 [26.1–43.9]	29.3 [25.3–36.0]	.18
**Cause of ESRD**						
Diabetes mellitus	11 (55.0)	74 (44.3)	.48	4 (44.4)	65 (45.1)	>.99
Hypertension	16 (80.0)	110 (65.9)	.31	6 (66.7)	98 (68.1)	>.99
Other etiologies^b^	3 (15.0)	42 (25.2)	.41	1 (11.1)	35 (24.3)	.69
**Comorbidities**						
Hypertension	20 (100.0)	157 (94.0)	.60	9 (100.0)	137 (95.1)	>.99
Diabetes mellitus	12 (60.0)	93 (55.7)	.81	4 (44.4)	82 (56.9)	.51
Cerebrovascular disease or accident	11 (55.0)	94 (56.3)	>.99	6 (66.7)	80 (55.6)	.73
Neurological disease (excluding CVA)	2 (10.0)	7 (4.2)	.25	1 (11.1)	6 (4.2)	.35
Blood disorder	1 (7.1)	3 (1.7)	.40	0 (0.0)	3 (2.1)	.53
Solid-organ transplant	1 (5.0)	12 (7.2)	>.99	0 (0.0)	12 (8.3)	>.99
Autoimmune conditions (including systemic lupus erythematosus)	0 (0.0)	12 (7.2)	.37	0 (0.0)	10 (6.9)	>.99
Other comorbidity^c^	5 (25.0)	50 (29.9)	.80	1 (11.1)	43 (29.9)	.45
**SARS-CoV-2 prevention behaviors**						
**Reported social distancing at dialysis center (% adherence)**			.42			>.99
All the time	13 (65.0)	125 (74.9))		6 (66.7)	97 (67.4)	
Less than all the time	7 (35.0)	42 (25.1)		3 (33.3)	47 (32.6)	
**Reported mask use at dialysis centers**			>.99			>.99
All the time	19 (95.0)	157 (94.0)		8 (88.9)	130 (90.3)	
Less than all the time	1 (5.0)	10 (6.0)		1 (11.1)	14 (9.7)	
**Reported hand hygiene at dialysis center prior to sitting on dialysis chair**			.40			>.99
All the time	14 (70.0)	131 (78.4)		5 (55.6)	87 (60.4)	
Less than all the time	6 (30.0)	36 (21.6)		4 (44.4)	57 (39.6)	
**Reported social distancing during daily life outside of home**			.003			.41
All the time	10 (50.0)	136 (81.4)		6 (66.7)	114 (79.2)	
Less than all the time	10 (50.0)	31 (18.6)		3 (33.3)	30 (20.8)	
**Reported mask use during daily life outside of home**			>.99			.33
All the time	18 (90.0)	151 (90.4)		7 (77.8)	126 (87.5)	
Less than all the time	2 (10.0)	16 (9.6)		2 (22.2)	18 (12.5)	
**Reported change in hand washing habits during daily life**			.61			>.99
Yes, wash hands more often	15 (75.0)	111 (66.5)		5 (55.6)	86 (59.7)	
No, wash hands about the same amount as before	5 (25.0)	56 (33.5)		4 (44.4)	58 (40.3)	
Attended a gathering with more than 10 people in prior 3 months	6 (30.0)	75 (44.9)	.24	3 (33.3)	60 (41.7)	.74
No. of gatherings attended, median [IQR]^d^	1 [1–1]	2 [1–4]	.10	2 [2–12]	2 [1–5]	.36
Visit to other health facilities (doctor offices, dentist offices) in prior 3 mo	11 (55.0)	124 (74.3)	.11	5 (55.6)	93 (64.6)	.72
No. of doctor/dentist appointments, median [IQR]^d^	1 [1–5]	2 [1–4]	.38	3 [2–3]	3 [1–4]	.81
Visits to public spaces for daily activities in prior 3 mo	19 (95.0)	138 (82.6)	.21	5 (55.6)	116 (80.6)	.09
No. of visits to public locations, median [IQR]^d^	19 [4–46]	15 [6–31]	.55	3 [1–9]	18.5 [7–41]	.041
**SARS-CoV-2 exposures**						
Travel outside of the St Louis area	1 (5.0)	12 (7.2)	>.99	0 (0.0)	9 (6.3)	.07
Household member tested for SARS-CoV-2	13 (65.0)	55 (32.9)	.007	3 (33.3)	43 (29.9)	>.99
Household member tested positive for or diagnosed with SARS-CoV-2	8 (40.0)	7 (4.2)	<.001	1 (11.1)	4 (2.8)	.26
Household member told by health department to stay home due to SARS-CoV-2 exposure	8 (40.0)	7 (4.2)	<.001	3 (33.3)	3 (2.1)	.003
Household member hospitalized with SARS-CoV-2	8 (40.0)	4 (2.4)	<.001	1 (11.1)	0 (0.0)	.06
Extended family/friend tested positive for or diagnosed with SARS-CoV-2	3 (15.0)	14 (8.4)	.40	0 (0.0)	9 (6.3)	>.99
Extended family/friend hospitalized with SARS-CoV-2	5 (25.0)	15 (9.0)	.045	0 (0.0)	17 (11.8)	.60
Extended family/friend died from SARS-CoV-2	3 (15.0)	14 (8.4)	.40	1 (11.1)	10 (6.9)	.50

Note. ND, not determined; BMI, body mass index; ESRD, end-stage renal disease; PKD, polycystic kidney disease; CAD, coronary artery disease; CVA, cerebrovascular accident; HF, heart failure; HD, hemodialysis; IQR, interquartile range.

a Defined as a positive PCR and/or serology for SARS-CoV-2.

b Other etiologies include glomerulonephritis, lupus nephritis, PKD, other.

c Other comorbidities include active malignancy, cirrhosis, current smoker, HIV, lung disease.

d Restricted to patients who reported attended a gathering with <10 people, a visit to a health facility or a visit to a public location.

Potential transmission events in the HD centers were examined (Supplementary Fig. 1). Most cases did not appear to be temporally or spatially related to any other SARS-CoV-2 cases. One shift at facility A had 3 positive patients who reported generally sitting in the same pod; 1 patient had a positive PCR result in November and 2 additional patients were IgG positive in December without any known SARS-CoV-2 exposures.

## Discussion

Studies examining risk factors associated with SARS-CoV-2 infection among chronic HD patients and HCP working in HD units using repeated surveys are lacking. In our prior publication, several risk factors at baseline were associated with SARS-CoV-2 infection, including SARS-CoV-2 infection among immediate family members or friends, residence in a long-term care facility, poor adherence to face mask use, and travel outside the local metropolitan area.^11^ However, in the subsequent surveys, the only risk factor that remained associated with SARS-CoV-2 infection among chronic HD patients was having a household member with SARS-CoV-2 infection. Among HCP, the only risk factor associated with SARS-CoV-2 infection was having a household member with SARS-CoV-2 infection.

There appeared to be no patient-to-patient or patient-to-staff transmission in the dialysis units; however, we did not perform whole-genome sequencing to confirm this. In one facility, 2 patients were identified as IgG positive in without known SARS-CoV-2 exposures in the month after an infected patient was dialyzed in the same pod. Because it is not known when the 2 IgG-positive patients were exposed to SARS-CoV-2, no clear relationship between these cases could be discerned. The 3 HD units implemented the Centers for Disease Control and Prevention (CDC) SARS-CoV-2 infection prevention measures in March 2020.^12^ Previous studies reported that implementation of infection prevention measures (eg, a universal mask policy) in HD units were associated with reduced risk of SARS-CoV-2 infection.^9,13^ Our results are consistent with a previous study^9^ indicating that when recommended infection prevention measures are used in HD units, the risk of SARS-CoV-2 infection is dependent on exposures from contacts in patient homes, especially when the community burden of SARS-CoV-2 is high.

Our study had several limitations. Due to the small number of cases, we could not perform multivariate analysis to identify independent risk factors for SARS-CoV-2 infection. Survey responses may be subject to recall bias. Finally, our study was performed when most patients had not received the SARS-CoV-2 vaccine (only 33% of patients had received at least 1 dose of vaccine at the 6-month survey) and prior to the emergence of the SARS-CoV-2 o(omicron) variant. Thus, our results may not be generalizable to new SARS-CoV-2 variants that emerged after the study period or among vaccinated chronic HD patients.

In this longitudinal survey study in 3 outpatient HD units that implemented CDC SARS-CoV-2 infection prevention measures, the risk of SARS-CoV-2 infection among HD patients and HCPs was consistently associated with a household member having SARS-CoV-2 infection.

## Supplementary material

For supplementary material accompanying this paper visit https://doi.org/10.1017/ash.2022.269. click here to view supplementary material
