# Supplementary material for: Longitudinal analysis of risk factors associated with severe acute respiratory coronavirus virus 2 (SARS-CoV-2) infection among hemodialysis patients and healthcare personnel in outpatient hemodialysis centers
Source: Antimicrob Steward Healthc Epidemiol. 2022 Jul 21;2(1):e125. doi: 10.1017/ash.2022.269 (PMC9726589; doi:10.1017/ash.2022.269)

Supplementary Table 1: Outpatient hemodialysis facilities characteristics included in the study

	Facility A	Facility B	Facility C
Total number of HD patients	151	118	40
Number of enrolled HD patients at baseline	101	94	31
Total number of HCPs	34	33	12
Number of enrolled HCPs at baseline	20	12	7
Number of Pods	4	3	4
Total number of HD treatment chairs	32	24	16
Distance between chairs	>6 feet	4.8 feet for most chairs and 6 feet for few chairs	>6 feet

HD- Hemodialysis

HCPs- Healthcare Personnel

Supplementary Table 2: Distribution of HD patients with SARS-CoV-2 infection at baseline, three months and six months

Baseline (N=227)				
	**SARS-CoV-2 PCR +**	**SARS-CoV-2 PCR -**	**No PCR test**	**Total**
**Serology test +**	11	3	5	19
**Serology test -**	3	115	90	208
3 months (N=187)* - excluding patients with + PCR or serology test at baseline				
	**SARS-CoV-2 PCR +**	**SARS-CoV-2 PCR -**	**No PCR test**	**Total**
**Serology test +**	8	1	5	14
**Serology test -**	6	60	107	173
6 months (N=153)** - excluding patients with + PCR or serology test at baseline or at 3 months				
	**SARS-CoV-2 PCR +**	**SARS-CoV-2 PCR -**	**No PCR test**	**Total**
**Serology test +**	5	0	4	9
**Serology test -**	0	32	112	144

*18 patients lost to follow-up between baseline and 3 months

**14 patients dropped out between 3 months and 6 months

Supplementary Figure 1: Distribution of SARS-CoV-2 infected cases by hemodialysis facility, pod, and shift

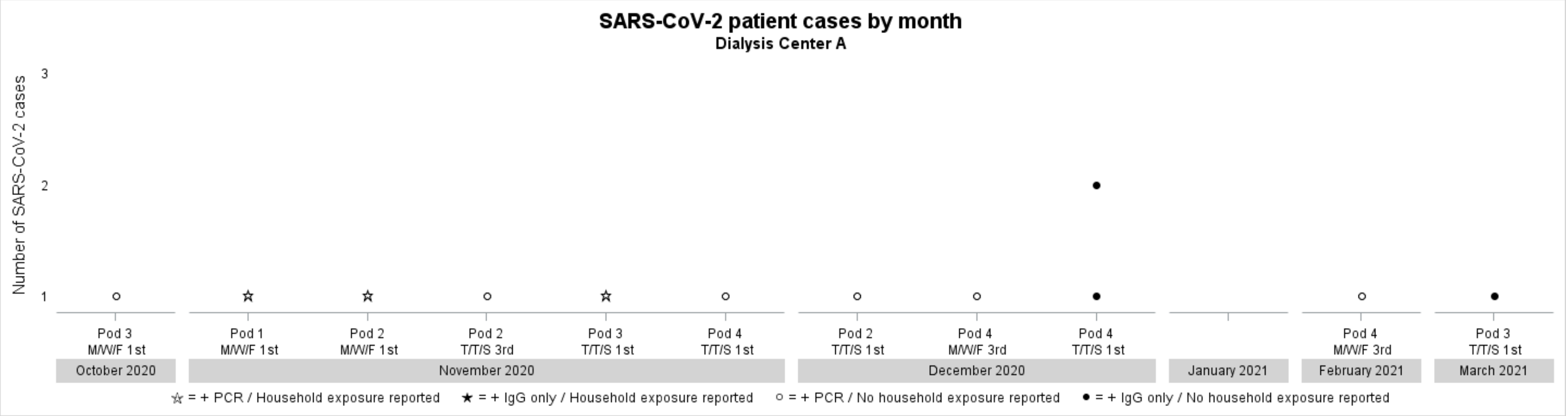

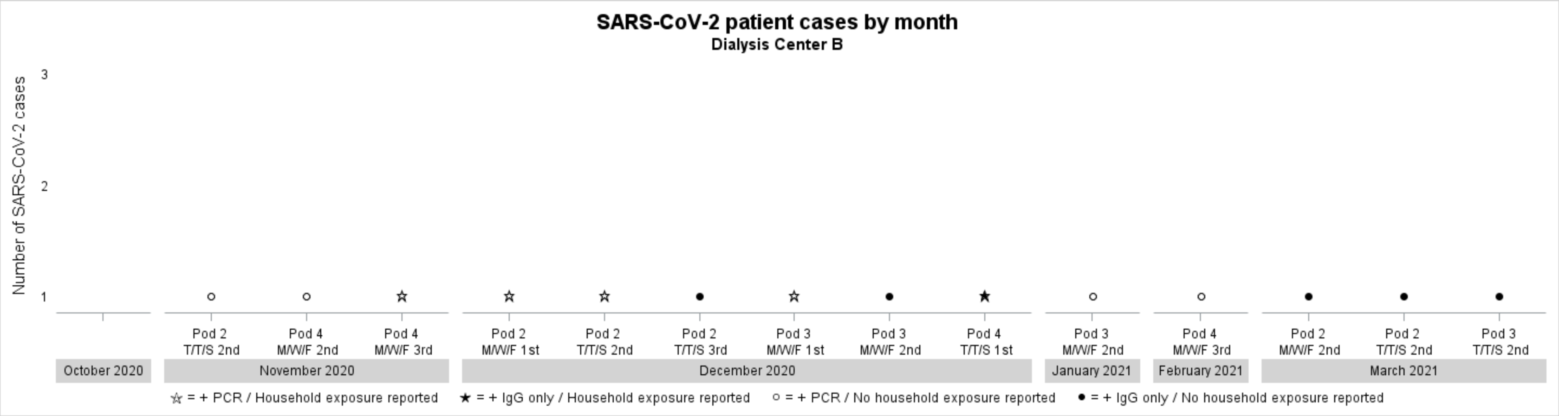

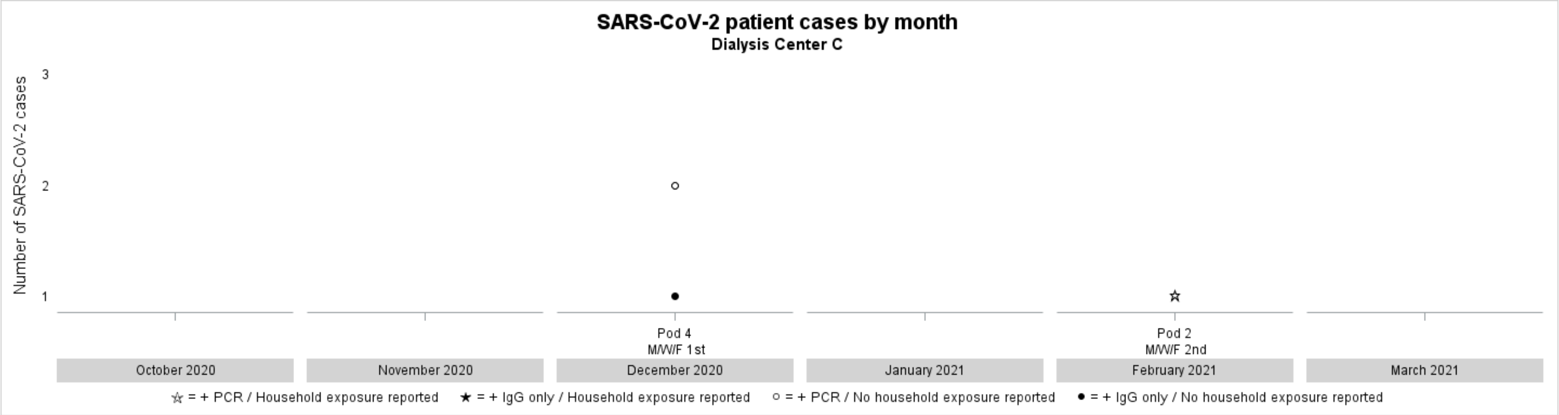

Supplement: Supplementary file 1 [file S2732494X22002698sup001.pdf]
